# A model to estimate the cost of the National Essential Public Health Services Package in Beijing, China

**DOI:** 10.1186/s12913-015-0902-4

**Published:** 2015-06-06

**Authors:** Delu Yin, Sabrina T. Wong, Wei Chen, Qianqian Xin, Lihong Wang, Mingming Cui, Tao Yin, Ruili Li, Xiaoguo Zheng, Huiming Yang, Juanjuan Yu, Bowen Chen, Weizhong Yang

**Affiliations:** Capital Institute of Pediatrics, 2 YaBao Road, #328, ChaoYang District, Beijing, 100020 China; Chinese Center for Disease Control and Prevention, 155 ChangBai Road, ChangPing District, Beijing, 102206 China; Community Health Association of China, Beijing, China; University of British Columbia School of Nursing and Centre for Health Services and Policy Research, 6190 Agronomy Road, #302, Vancouver, BC V6T 1Z3 Canada; Beijing University of Chinese Medicine, 11 BeiSanhuan East Road, ChaoYang District, Beijing, 100029 China

**Keywords:** Cost estimate, National essential public health services, Public health, Primary healthcare, China

## Abstract

**Background:**

In order to address several health challenges, the Chinese government issued the National Essential Public Health Services Package (NEPHSP) in 2009. In China’s large cities, the lack of funding for community health centers and consequent lack of comprehensive services and high quality care has become a major challenge. However, no study has been carried out to estimate the cost of delivering the services in the package. This project was to develop a cost estimation approach appropriate to the context and use it to calculate the cost of the NEPHSP in Beijing in 2011.

**Methods:**

By adjusting models of cost analysis of primary health care and workload indicators of staffing need developed by the World Health Organization, a model was developed to estimate the cost of the services in the package through an intensive interactive process. A total of 17 community health centers from eight administrative districts in Beijing were selected. Their service volume and expenditure data in 2010 were used to evaluate the costs of providing the NEPHSP in Beijing based on the applied model.

**Results:**

The total workload of all types of primary health care in 17 sample centers was equivalent to the workload requirement for 14,056,402 standard clinic visits. The total expenditure of the 17 sample centers was 26,329,357.62 USD in 2010. The cost of the workload requirement of one standard clinic visit was 1.87 USD. The workload of the NEPHSP was equivalent to 5,514,777 standard clinic visits (39.23 % of the total workload). The model suggests that the cost of the package in Beijing was 7.95 USD per capita in 2010. The cost of the NEPHSP in urban areas was lower than suburban areas: 7.31 and 8.65 USD respectively.

**Conclusions:**

The average investment of 3.97 USD per capita in NEPHSP was lower than the amount needed to meet its running costs. NEPHSP in Beijing is therefore underfunded. Additional investment is needed, and a dynamic cost estimate mechanism should be introduced to ensure services remain adequately funded.

## Background

In the past few decades, the public health system in China rapidly improved. The average life expectancy increased from 71.4 to 74.8 years between 2000 and 2010 [1]. The maternal mortality rate was reduced from 51.3 to 26.1 per 100,000 population, and the infant mortality rate from 29.2 to 12.1 per 1,000 live births between 2002 and 2011 [2–5]. Even with such achievements, China faces new public health challenges of hypertension, obesity, diabetes, and other non-communicable diseases [6, 7]. To address these issues and curb use of more costly secondary and tertiary care, early prevention and intervention programs have been under development since 2009.

One such program is the National Essential Public Health Services Package (NEPHSP). It aims to provide the same public health services regardless of geographic area and expand coverage of essential public health programs to all residents of China [8]. The program includes 10 services (see Table 1) that are mainly focused on children aged 0 to 36 months, pregnant women, those aged over 65 and patients with chronic conditions and mental health issues [9, 10].

**Table 1:** Types of services included in the NEPHSP in 2009

Types	Details
Health records management	Establishing and updating health records and health information for residents who have lived in the area for more than 6 months
Health education	Health education and publications about unhealthy life style, risk factors, and diseases to all the residents in the area
Health services for children aged 0 to 36 months	Home visits to newborn infants, physical examinations for children, and health education and guidance to parents of children aged 0 to36 months
Maternal health services	Maternity care before and after delivery, and a post-natal physical examination 42 days after delivery
Older people’s health services	Physical examinations, health advice, guidance and intervention for all those aged over 65
Immunizations	Routine immunizations for children aged 0 to 3 and for vulnerable older people
Infectious disease reporting and treatment	Registering, reporting, and managing patients with (suspected) notifiable diseases and their close contacts
Health services for patients with hypertension	Establishing health records, screening and following-up and systematic physical examinations of anyone aged over 35 with hypertension
Services for patients with type II diabetes	Establishing health records, screening and following-up and systematic physical examinations for anyone aged over 35 with Type II diabetes
Services for patients with severe mental illness	Establishing health records and providing follow-up services for any patients with severe mental illness who are living at home

(Source: National Essential Public Health Services Guidelines, 2009)

China’s community-based primary healthcare (PHC) facilities are designed to deliver comprehensive PHC services, from family planning to rehabilitation, and the NEPHSP. These universally-accessible [11] PHC facilities include village and township clinics in rural areas and community health service facilities in urban areas. The package of services available to all residents was originally funded by local, provincial, and national governments at 2.38 USD per person [12]. However, this funding was insufficient, leading to services that were not comprehensive, low quality of care and an insufficient volume of services delivered through PHC facilities [13–15]. In China’s large cities, the lack of funding for community health service facilities and consequent lack of comprehensive services and high quality care [16–18] has become a major challenge. Beijing, the home of over 20 million people, is not exempt from these challenges.

However, little information exists on how to appropriately cost services provided by the PHC facilities [19–21]. Historically, the ladder-sharing method has been used by the Chinese government to examine costs of health services [22]. The basic steps include collecting the total cost of the facilities, determining the direct costing departments and indirect costing departments and defining the costs of each, then distributing the indirect costs to the direct-cost departments and getting their total costs. The final step is calculating the costs of the targeted health services by workload in the direct-costing departments. However, this method cannot be used in PHC settings because of several inherent limitations. First, PHC includes various categories and types of services that can be delivered by a single healthcare professional. It is therefore hard to allocate indirect cost to different departments [23]. Second, the ladder-sharing method requires relatively accurate data for costing [24, 25], but PHC facilities do not have advanced health information systems or financial management systems. Finally, it is impossible for the ladder-sharing method to estimate investment need when new services are added to the package, which happens frequently as China’s fiscal capacity increases. The purpose of this project was to develop a cost estimation approach appropriate to the PHC context and use it to calculate the cost of the NEPHSP in Beijing.

## Methods

### Setting

Beijing, in northern China, is China’s capital city, and home to over 20,000,000 people. Most PHC services are delivered through publicly-funded community health centers (CHCs) and smaller, affiliated, community health stations. There are 327 community health facilities located throughout Beijing, which provide residents with a comprehensive set of PHC services delivered by medical, nursing, and paramedical teams. Community health facilities also provide traditional Chinese medicine.

We conducted a secondary analysis of data collected from 17 randomly-selected CHCs located in eight administrative districts (Xicheng, Haidian, Chaoyang, Mentougou, Huairou, Miyun, Pinggu and Tongzhou). Seven of these centers were in urban districts and 10 in suburban areas. No national information or reporting system for the community-based PHC system currently exists [12]. However, all eligible sites had computerized electronic medical records. The Ethics Committee of the Capital Institute of Pediatrics approved the study. All the participants in the selected centers provided written informed consent, and all the patient information was anonymized and de-identified prior to analysis.

### Procedures for data collection

Data on costs were collected as part of a larger study. CHC managers were trained in how to collect the necessary data. A standardized data collection tool was used to collect basic information, and the expenditure and volume of each type of PHC services, including the 10 types in the NEPHSP. Data were collected over a 3-week period. Two separate researchers conducted site visits to oversee the data collection.

Expenditure in the sampled CHCs was classified based on the resource inputs used for the service [26, 27]. These included human resources, material expenditure and public expenses, as set out by the government. At the time, the CHCs in Beijing had separate management of income and expenditure, so all income was from public sources. Human resource expenditure included basic salaries, allowances, bonuses, social insurance, housing accumulation funds, purchasing subsidies, living subsidies, and medical expenses. Material expenditure included medicines, health materials and low-valued consumables. Public expenses expenditure included printing, water, electricity, mail services, transportation, travel expenses, and meeting and hospitality costs.

### Model

Based on methods used by the World Health Organization in a manual on cost analysis of PHC [28, 29], four steps were developed to estimate the costs of the NEPHSP in Beijing: 1) determine the standard service protocols of all types of PHC services; 2) define the workload indicators needed for a set of standard activities for services, and their equivalent value (EV) compared with a standard clinic visit; 3) calculate the average cost of one EV; 4) calculate the cost of NEPHSP per capita.

#### Step 1: Determining the standard service protocols

All types of PHC services providing by the sampled CHCs were investigated. In total, there were 65 types of PHC services deemed necessary for inclusion in the standard service protocols, including medical, nursing, paramedical, the NEPHSP and other public health services, and auxiliary examinations. The main types are listed in Table 2. Only 10 of the 65 were included in the 2009 NEPHSP guidelines. The remaining 55 services were defined in line with the 2007 Beijing technical specification for primary health services [30], and were similar across urban and suburban areas.

**Table 2:** Workload and EV of the main PHC services compared with a standard clinic visit

Categories	Types	Urban		Suburban	
		Workload	Mean EV	Workload	Mean EV
Essential medical services	Clinic visit (per visit)	15.00	1.00	15.00	1.00
	Emergency (per visit)	40.35	2.69	52.50	3.50
	Home visit (per visit)	60.00	4.00	60.00	4.00
	Inpatient bed day	100.05	6.67	180.00	12.00
	Rehabilitation clinic (per outpatient visit)	30.00	2.00	30.00	2.00
Nursing services	Intravenous injection	7.50	0.50	6.00	0.40
	Intravenous infusion	12.00	0.80	11.70	0.78
	Intravenous injection, venous blood	9.30	0.62	9.45	0.63
	Catheterization	28.95	1.93	19.50	1.30
	Providing prescription (western medicine)	4.50	0.30	6.00	0.40
Pharmacy service	Providing medication to meet a prescription (per prescription)	19.95	1.33	19.95	1.33
	Advanced pharmacy work, including detailed dosage calculations (per prescription)	63.60	4.24	60.00	4.00
Auxiliary examination service	Rapid blood sugar test	4.50	0.30	4.65	0.31
	Blood, urine, feces test (per test)	9.00	0.60	9.15	0.61
	Biochemical test (per test)	30.00	2.00	27.45	1.83
	Electrocardiogram (per test)	11.25	0.75	10.05	0.67
	B-mode Ultrasonography (per test)	19.95	1.33	19.95	1.33
NEPHSP	Health records management service (per person year)	1,019.25	67.95	972.00	64.80
	Health education service (per center)	47,076.00	3,138.40	34,650.00	2,310.00
	Health services for children aged 0 to 36 months (per person year)	173.40	11.56	203.40	13.56
	Maternal health services (per person year)	210.00	14.00	262.50	17.50
	Older people’s health services (per person year)	60.00	4.00	49.95	3.33
	Immunizations (per visit)	25.05	1.67	15.45	1.03
	Infectious disease reporting and treatment (per time)	5,913.6	394.24	4,979.40	331.96
	Patients with hypertension (per person year)	190.05	12.67	152.55	10.17
	Patients with type II diabetes (per person year)	190.05	12.67	147.45	9.83
	Patients with severe mental illness (per person year)	472.50	31.50	420.00	28.00

### Step 2: Calculating the workload indicator and the equivalent value (EV) of each PHC service

#### Step 2.1: Workload indicator of each PHC service

In order to create the workload indicator (person-time) of each PHC service, a multi-stage iterative feedback and revision process were conducted. A series of four meetings were held with participants (n = 72). They were invited to attend these meetings based on their knowledge and expertise about PHC and their region (urban or suburban). Stakeholders included community health service managers (n = 36), family physicians (n = 18), nurses (n = 10), and public health workers (n = 8). During the meetings, participants discussed the person and amount of time required for each PHC service, and discussed the workload assigned to each service in the standard service protocols. They also discussed suggested modifications. Since different time periods are needed for the same type of service in urban and suburban PHC facilities, because of the different population density and delivery model, two sets of specific workload indicators were created for each PHC service.

To test these workload indicators, eight CHCs (four urban and four suburban) were randomly selected from the sample of 17 to participate in direct observations. Five research assistants were trained to observe the services and record the length of time for each PHC service and the number of health workers involved. Direct observation took place over a period of three continuous days in each CHC. Face-to-face interviews were conducted to determine the usual time and the required number of health workers for services for which these details could not be recorded during the period of direct observation.

The workload indicators were modified based on the direct observation and interviews. Group interviews with the staff at the eight centers (n = 32) were then conducted to test the workload indicators. Family physicians (n = 8), nurses (n = 8), public health workers (n = 8), and other health professionals (n = 8) participated.

#### Step 2.2: Equivalent value of each PHC service

To ensure that different types of services can be directly compared, a “standard clinic visit” was introduced as a benchmark to gauge the necessary people and time required (workload indicator) for the other services. A standard clinic visit was defined a family physician consulting with one patient for 15 min [31]. The workload indicator of “a standard clinic visit” was defined as one equivalent value (EV). Equivalent values of all other PHC services were then calculated as their workload indicator (step 2.1) compared with the standard clinic visit. For example, a home visit may include the time taken to travel to and from the patient’s home, and to administer medication. The workload indicator of one home visit was 60.00 person-time in both urban and suburban areas, so its EV was 4 (60/15). The EV of each PHC service in urban and suburban areas was defined separately and is shown in Table 2.

#### Step 3: Calculating the cost of one EV (a standard clinic visit)

The full expenditure and the volume of each PHC service in the 17 sampled CHCs were investigated. The volume was multiplied by the EV of each PHC service, and these figures were added together to get the total EVs of the 65 types of PHC service across the sampled CHCs. We used the following formula to obtain the cost of one EV (a standard clinic visit):

The cost of one EV = total expenditure in all the sampled CHCs /∑ volume × EV of each PHC service in each sampled CHCs.

### Step 4: Calculating the cost of the NEPHSP per capita

#### Step 4.1: Calculating the total workload of the NEPHSP and its total costs

The NEPHSP included 10 types of services. The volume of these 10 types in the sampled CHCs were multiplied by their EV, and then were added together to produce the total workload of the NEPHSP across all sites. The total EV of the NEPHSP was then multiplied by the cost of one EV to give the total cost of the NEPHSP across the sampled CHCs.

#### Step 4.2: Calculating the cost of the NEPHSP per capita

To calculate the cost of the NEPHSP per capita, the total population served by the sampled CHCs was taken from the standardized data collection tool. The population used was those resident for at least 6 months, and was reported by the sample CHCs to the local governmental Statistical Bureau. The following formula was used to compute the cost of the NEPHSP per capita.

The cost of the NEPHSP per capita = total cost of the NEPHSP across the sampled CHCs/total population served by the sampled CHCs.

## Results

### Sample CHCs and their expenditure

On average, each sample CHC had an average of 123 employees on the payroll (2,094 employees in 17 centers), serving on average a community of 76,259 people (a total of 1,296,403 people). It had an annual expenditure of 1.55 million USD (a total of 26,329,357.62 USD). The CHCs in urban areas had more employees, served more people and had more annual expenditure than suburban areas (see Table 3).

**Table 3:** Basic information for sampled community health centers

	Urban (n = 7)	Suburban (n = 10)	Both (n = 17)
Average employees per center	134.00	116.00	123.00
Average population served*	117,687.00	47,260.00	76,259.00
Total annual expenditure (USD)	12,756,127.14	13,573,230.79	26,329,357.62
Human resources expenditure (USD)	8,337,175.71	9,099,476.35	17,436,651.90
Material expenditure (USD)	1,038,666.67	1,532,670.48	2,571,336.98
Public funds expenditure (USD)	3,380,284.76	2,941,083.97	6,321,368.73

*The population used here was those resident for at least 6 months, and was reported by the sample center to the local governmental Statistical Bureau

### Mean EV of PHC services (including NEPHSP) and the total workload

The EV of the standard clinic visit was 1.00. Table 2 shows the mean EV of some main types of PHC services including NEPHSP. The EV in urban areas was larger than in suburban areas. The reasons for this include population density, and delivery model.

The total EVs of the 17 sampled CHCs including basic medical services, nursing, pharmacy, auxiliary examinations, and the NEPHSP in 2010 was 14,056,402. The EVs of basic medical services and the NEPHSP accounted for the majority, 32.10 % and 39.23 % respectively. The average EVs for each center were 826,847 (see Table 4).

**Table 4:** EV of the PHC services in 17 sampled community health centers

Categories	Urban		Suburban		Both	
Basic medical service	1,957,673	26.30 %	2,553,832	38.62 %	4,511,505	32.10 %
Nursing service	311,082	4.18 %	226,897	3.43 %	537,979	3.83 %
Pharmacy service	663,543	8.91 %	621,532	9.40 %	1,285,075	9.14 %
Auxiliary examination service	245,811	3.30 %	555,615	8.40 %	801,426	5.70 %
NEPHSP	3,520,967	47.30 %	1,993,810	30.15 %	5,514,777	39.23 %
*Other services	744,342	10.00 %	661,298	10.00 %	1,405,640	10.00 %
Total	7,443,417	100.00 %	6,612,985	100.00 %	14,056,402	100.00 %

*These services cannot be included in any category above. Group interviews suggested their workload could account for 10 % of the total

### Cost of one EV

The full expenditure and volume of each PHC service in the 17 sampled CHCs are shown in Table 3. The total workload of 65 types of PHC services in the 17 sampled CHCs was 14,056,402 EVs. Table 3 shows that the total expenditure in 2010 was 26,329,357.62 USD. It was therefore estimated that the average cost of one EV was 1.87 USD. The cost of one EV in urban areas was higher than that in suburban areas (see Table 5).

**Table 5:** Cost of one EV (USD)

Cost items	Urban	Suburban	Total
Human resources costs	1.12	1.38	1.24
Materials costs	0.14	0.23	0.18
Public funds	0.45	0.44	0.45
Total	1.71	2.05	1.87

### Cost of the NEPHSP per capita

As shown in Table 4, the total EVs of the NEPHSP in the 17 sampled CHCs were 5,514,777, which accounted for 39.23 % of the total EVs (urban: 3,520,967, 47.30 %; suburban: 1,993,810, 30.15 %). The cost of one EV was 1.87 USD. The total cost of the NEPHSP across all the sampled CHCs was 10,312,633 USD in 2010. The total number of served people was 1,296,408, giving an average cost per capita of 7.95 USD. The cost per capita in urban areas was lower than suburban areas (see Table 6).

**Table 6:** Cost of the NEPHSP per capita (USD)

Cost items	Urban	Suburban	Total
Human resources costs	4.79	5.82	5.27
Materials costs	0.60	0.97	0.76
Public funds	1.92	1.86	1.92
Total	7.31	8.65	7.95

## Discussion

In 2009, 2.38 USD per capita was allocated by the Chinese central government to cover the operating costs of the PHC package. This was increased to 3.97 USD in 2011 [9, 10], but is still much lower than the amount needed to meet the standard running costs, which were about 7.95 USD per capita in Beijing. This study confirmed that the NEPHSP program in Beijing is underfunded [13–15].

The population density in suburban areas is lower and these areas are more mountainous, thus it is harder to provide the same service to suburban residents, especially with home visits included. However, the average number of employees in the sample suburban centers was lower (Table 2). The labor costs per output and material costs were therefore higher than for urban areas. The public funding allocation is related to the population served, so is the same per capita for urban and suburban areas. This made the average cost per capita in suburban areas higher than in urban areas. It is possible that there was a certain economy of scale at work in urban areas. It may therefore be possible to lower the cost of services in suburban areas by merging some of the providers to serve a larger population. However, this will weaken accessibility. Further studies are needed to establish whether this would be feasible.

Currently, NEPHSP is one of the most important health care systems in Beijing [32]. Our study revealed that NEPHSP accounted for almost 40 % of the total PHC provision. Although government-funded, PHC in China remains underfunded and understaffed [33, 34]. With the expanding of NEPHSP, the development of PHC will be hindered if government investment in NEPHSP remains lower than costs. To provide high quality NEPHSP services, and to address the potential challenges, the Chinese government needs to take immediate action to increase investment in PHC.

Even if the government increased financial input to meet the real costs, any new allocation would soon be inadequate as the costs associated with care would continue to rise. A dynamic cost estimate mechanism should therefore be established, to keep abreast of changes in primary health service regulations, personnel allocations, commodity prices, salaries, and other costs. With the further improvement of the PHC information system, and by using this new model, the costs could be tracked in real time to obtain accurate and timely results. Funding inputs could then be adjusted to support the development of PHC services.

The determination of EV needs only to take number of medical personnel and time into consideration. This may potentially reduce the degree of technical difficulty in estimating costs. In 2013, the government increased the financial investment of NEPHSP, and adjusted the service requirements [35]. As new types of services were added to the package in the future, the cost estimate model used in this study could be adjusted onsite by simulation to determine the number of medical personnel and the amount of time needed. The result could be helpful to the government in assessing the funding requirements of the added service(s).

We note that this study has certain limitations. First, the estimate of medical personnel and time needed were based on service protocols, rather than the actual process or content. The cost calculated was therefore only theoretical. The validity of the method to estimate cost of NEPHSP needs to be tested further in practice. Second, we would have preferred to use national PHC guidelines for types other than NEPHSP as the standard service protocols to define their EV. However, these protocols were not widely-used or tested for validity [36].

## Conclusions

Our results show that it is possible to establish a mechanism for estimating the cost of primary healthcare services in Beijing. While more research is needed to validate the method, the average funding of 3.97 USD per capita in NEPHSP was lower than the amount estimated to meet the standard running costs in Beijing. This suggests that the service is underfunded. We suggest that a dynamic cost estimate mechanism should be introduced to ensure funding remains sufficient.

## Abbreviations

NEPHSP: National Essential Public Health Services Package
PHC: Primary healthcare
CHCs: Community health centers
EV: Equivalent value
